# Molecular Dynamics Simulation of the Interfacial Shear Properties between Thermoplastic Polyurethane and Functionalized Graphene Sheet

**DOI:** 10.3390/polym14225032

**Published:** 2022-11-20

**Authors:** Yuyang Wang, Guangping Zou, Junpeng Liu

**Affiliations:** 1 College of Aerospace and Civil Engineering, Harbin Engineering University, Harbin 150001, China; 2 Science and Technology on Combustion, Internal Flow and Thermostructure Laboratory, Northwestern Polytechnical University, Xi’an 710071, China

**Keywords:** graphene, thermoplastic polyurethane, interfacial shear properties, molecular dynamics simulation

## Abstract

In this study, the effect of the type and content of functional groups on the interfacial shear properties of a functionalized graphene sheet (FGS)/thermoplastic polyurethane (TPU) nanocomposite are investigated by molecular dynamics (MD) simulations. The maximum pull-out force and separation energy were used to characterize the interfacial strength of the FGS/TPU nanocomposite in sliding mode. To find out how the type and content of functional groups affect the interfacial shear properties of the TPU/FGS system from an atomic view, the details of interactions between FGS and TPU were characterized. Based on the results, stronger interfacial shear properties of the TPU/FGS system can be achieved by adding the carboxyl group or hydroxyl group on the surface of graphene than that between TPU and FGS modified by the amine group or epoxy group, because of the strong interaction of electrostatic forces and H-bonds. In addition, interfacial shear properties can also be enhanced by increasing the content of functional groups modified on the surface of graphene.

## 1. Introduction

As a typical linear alternating block copolymer, thermoplastic polyurethane (TPU) shows superior performance in terms of impact resistance, shock, vibration reduction, and wear resistance due to the morphology of the micro-phase separation at the mesoscopic scale. Thus, TPUs have been widely applied in aerospace, ship building, the coating of armor protection, and civil engineering. Nevertheless, as the rapid development of science technology leads to the requirement of higher properties for application materials, pure TPUs are difficult to apply in advanced applications due to the exposed shortcomings of the thermomechanical properties (e.g., low stiffness, poor tensile strength, and poor thermal stability) [1,2].

Recently, it was found that filling carbon nanofillers into the polymer matrix has been widely considered an efficient approach to enhance the mechanical properties of neat polymer materials [3,4,5,6]. Among them, the graphene sheet is gaining more attention because it has a higher surface-to-volume ratio and exhibits more flexibility, which is attributed to the unique two-dimensional structural characteristic [7]. In addition, the higher surface-to-volume ratio determines the larger contact area between nanofillers and polymer chains, which results in the mechanical properties of the neat polymer system by the incorporation of graphene into the polymers being superior to that by the incorporation of CNTs. Wang et al. reported that the ultimate tensile strength of polyvinyl alcohol can be significantly improved by incorporating only 0.5 wt% of a graphene sheet [8]. Yasmin et al. found that the mechanical properties of polymethyl methacrylate (PMMA) and epoxy matrix can be substantially improved by adding small concentrations of graphene sheets [9]. Wang et al. reported that the addition a low fraction of 2.0% of a graphene sheet results in a 239% increase in the ultimate tensile strength and a 202% increase in Young’s modulus [10].

According to previous reports in the literature, the thermomechanical properties of polymer nanocomposites can be affected by the dispersion and alignment of graphene, the individual properties of the polymer matrix and filler, and the interaction properties between the polymer matrix and the filler. Among them, due to the interaction properties of graphene-based polymer nanocomposite systems directly determining the degree of load transfer between the polymer matrix and graphene, understanding and improving the interactions have been critical issues to be resolved. Khatir et al. studied the adsorption of lactate molecules on different graphene conformations, and they found that the absorption energy of the lactate molecule on ψ-graphene is much higher than that of graphene [11,12]. Li et al. studied the effect of defects on the interfacial mechanical properties of graphene/epoxy composites by MD simulations, and the results showed that the addition of Stone–Wales defects in graphene can effectively improve the mechanical properties of graphene/polymer nanocomposites [13]. Wang et al. conducted normal and transverse pull-out simulations to investigate the influence of ambient temperature and the hard-segment content of TPU on the interfacial properties of a TPU/graphene system [14]. Sahraei et al. applied MD simulations to investigate the effect of the number of graphene layers and the epoxy crosslinking density on the interfacial characterization of graphene/epoxy nanocomposites by comparing the interphase thickness and interfacial shear properties [15,16].

Recent studies have shown that the pristine graphene sheets incorporated into the polymer matrix tend to aggregate in the preparation process due to its high area-to-volume ratio by the van der Waals interaction, which results in negative effects on the enhancement of the mechanical properties of the graphene-based polymer nanocomposite. To achieve the homogenous dispersion of graphene into the polymer matrix, a method of modifying the surface of graphene with functional groups was proposed. Some researchers found that with the introduction of functional groups, graphene can be better dispersed in the polymer matrix [17,18], and it was also found that the mechanical properties of polymer composites can be significantly enhanced by filling the polymer with functionalized graphene. Pokharel et al. reported that the thermomechanical properties of polyurethane composites can be significantly enhanced by modifying functional groups with 2% content on the surface of graphene in the case of the same amount of graphene or graphene oxide filled into the TPU matrix [19]. Skountzos et al. found that the mechanical reinforcement of GO/poly(methyl methacrylate) (PMMA) can be achieved by loading 5.67 wt% of GO [20].

Therefore, due to the superior performance of functionalized graphene in improving the thermomechanical properties of polymer nanocomposites, many studies have also focused on the effect of graphene surface modification on the thermomechanical properties of polymer composites, and one of the key factors to be addressed is the interfacial mechanical properties between functionalized graphene and the polymer matrix.

However, due to the experimental process’s complexity and the measurement methods’ limitations, it is almost impossible to directly measure and evaluate the interfacial interaction between functionalized graphene and the polymer matrix directly, especially between functional groups and the polymer matrix. This may require tracing back to the mesoscopic scale or even the microscopic scale to study the interactions between the atoms of the two systems in real time. For this reason, many researchers have started to prefer molecular dynamics to study the interfacial mechanical properties between the polymer matrix and functionalized graphene [21,22,23]. Recently, a computational approach-based pull-out test was widely applied in analyzing the interfacial properties between the polymer matrix and graphene or functionalized graphene, which can evaluate the load transfer in the polymer/graphene system by estimating the interfacial shear properties, such as pull-out force or threshold shear strength. Pokharel et al. performed pull-out simulations to investigate the effect of functional groups on the interfacial properties of FGS/polylactide (PLA) nanocomposites [24]. Melro et al. studied the effect of the type, content, and distribution of functional groups on the interfacial properties of graphene and epoxy by characterizing interfacial shear strength and pull-out force based on pull-out simulations [25]. Park et al. combined MD simulations and the shear deformation model to characterize the interfacial shear properties between an FGS and epoxy resin by pull-out simulations [26]. Wang et al. investigated the effect of different functional groups modified on the graphene nanofiber on the mechanical properties of polyvinyl alcohol nanocomposites, and the MD results showed that increasing properly the aspect rations of functional graphene nanofibers can improve the mechanical properties of the polymer nanocomposite [27].

Although a considerable number of studies have been reported on the interfacial mechanical properties between polymers and functionalized graphene using MD simulation methods, there is still a lack of simulation research on investigating the interfacial mechanical properties of the FGS/TPU nanocomposite. In addition, we realized that although the interfacial binding strength can be directly characterized by pull-out simulations, the underlying mechanism affecting the interfacial properties of TPU/FGS nanocomposites is still unclear. In this study, we performed a pull-out simulation to investigate the interfacial shear properties between the TPU matrix and the FGS. The interfacial shear pull-out force and separation energy were calculated during the pull-out process. Furthermore, the interfacial interaction energy and hydrogen bonds between the TPU matrix and the FGS were analyzed to uncover the underlying mechanism.

## 2. Materials and Methods

### 2.1. PCFF Forcefield

The Large-scale Atomic/Molecular Massively Parallel Simulator (LAMMPS) [28] was used to perform MD simulations. The intra- and intermolecular interactions were described by applying the polymer-consistent force field (PCFF) [29], which can effectively evaluate the mechanical and thermal properties of graphene/polymer nanocomposites [30,31,32]. According to the PCFF forcefield, the total potential energy of the system is divided into three major terms, which can be expressed as follows:

(1) $$E_{total}=E_{valence}+E_{cross-term}+E_{nonbond},$$

where $E_{valence}\;$ denotes the contributions from each of the internal valence coordinates, which contains bond-stretching terms, bond-angle-bending terms, torsion-angle rotation terms, and out-of-plane interaction terms. $E_{cross-term}$ denotes the coupling or cross-terms between two internal coordinates, and $E_{nonbond}\;$ denotes the nonbonded interactions, which includes van der Waals (vdW) energy and Coulomb electrostatic energy.

(2) $$E_{valence}=E_{bond}+E_{angle}+E_{torsion}+E_{oop},$$

(3) $$E_{nonbond}=E_{vdW}+E_{Coulomb}$$

Herein, the expression for each component of the potential energy term is given as follows in detail:

(4) $$E_{bond}=\sum_{b}\left[k_{2}^{b}{\left(b-b_{0}\right)}^{2}+k_{3}^{b}{\left(b-b_{0}\right)}^{3}+k_{4}^{b}{\left(b-b_{0}\right)}^{4}\right],$$

(5) $$E_{angle}=\sum_{\theta }\left[k_{2}^{\theta }{\left(\theta -\theta_{0}\right)}^{2}+k_{2}^{\theta }{\left(\theta -\theta_{0}\right)}^{3}+k_{2}^{\theta }{\left(\theta -\theta_{0}\right)}^{4}\right]$$

(6) $$E_{torsion}=\sum_{\phi }\left[k_{1}^{\phi }\left[1-\cos\left(\phi -\phi_{1}^{0}\right)\right]+k_{2}^{\phi }\left[1-\cos\left(2\phi -\phi_{2}^{0}\right)\right]+\;k_{3}^{\phi }\left[1-\cos\left(3\phi -\phi_{3}^{0}\right)\right]\right]$$

(7) $$E_{oop}=\sum_{\chi }k_{\chi }\chi^{2}$$

(8) $$E_{bond-bond}=\sum_{b,b^{\prime }}k_{bb^{\prime }}\left(b-b_{0}\right)\left(b^{\prime }-b_{0}^{\prime }\right)$$

(9) $$E_{angle-angle}=\sum_{\theta ,\theta^{\prime }}k_{\theta \theta^{\prime }}\left(\theta -\theta_{0}\right)\left(\theta^{\prime }-\theta_{0}^{\prime }\right)$$

(10) $$E_{bond-angle}=\sum_{b,\theta }k_{b\theta }\left(b-b_{0}\right)\left(\theta -\theta_{0}\right)$$

(11) $$E_{end-bond-torsion}=\sum_{b,\phi }k_{b\phi }\left(b-b_{0}\right)\left[V_{1}\cos\phi +V_{2}\cos2\phi +V_{3}\cos3\phi \right]$$

(12) $$E_{middle-bond-torsion}=\sum_{b^{\prime },\phi }k_{b^{\prime }\phi }\left(b-b_{0}\right)\left(b^{\prime }-b_{0}^{\prime }\right)\left[F_{1}\cos\phi +F_{2}\cos2\phi +F_{3}\cos3\phi \right]$$

(13) $$E_{angle-torsion}=\sum_{\theta ,\phi }k_{\theta \phi }\left(\theta -\theta_{0}\right)\left[V_{1}\cos\phi +V_{2}\cos2\phi +V_{3}\cos3\phi \right]$$

(14) $$E_{angle-angle-torsion}=\sum_{\theta ,\theta^{\prime },\phi }k_{\theta \theta^{\prime }\phi }\left(\theta -\theta_{0}\right)\left(\theta^{\prime }-\theta_{0}^{\prime }\right)\cos\phi$$

(15) $$E_{vdW}=\sum \varepsilon_{ij}\left(2{\left(\frac{r_{0,ij}}{r_{ij}}\right)}^{9}-3{\left(\frac{r_{0,ij}}{r_{ij}}\right)}^{6}\right)$$

(16) $$E_{Coulomb}=\frac{1}{4\pi \varepsilon_{0}\varepsilon_{r}}\sum \frac{q_{i}q_{j}}{r_{ij}}$$

where the constants $k_{i}^{b}\left(i=2-4\right)$, $k_{i}^{\theta }\left(i=2-4\right)$, $k_{i}^{\phi }\left(i=1-3\right)$, and *k_χ_* denote the stiffness of bond, angle, torsion, and out-of-plane potential terms, respectively. In addition, *b* and *b*′ denote the bond length of two adjacent atoms. *θ* and *θ*′ denote the bond angle enclosed by two adjacent bonds. *Φ* denotes the dihedral torsion angle, and *χ* denotes the out-of-plane angle. $k_{bb^{\prime }}$, $k_{\theta \theta^{\prime }}$, $k_{b\theta }$, $k_{b\phi }$, $k_{b^{\prime }\phi }$, $k_{\theta \phi }$, and $k_{\theta \theta^{\prime }\phi }$ are the coefficients of the cross-term potential term fitted from quantum mechanics; *q* represents the electrostatic charge; and constant *ε* represents the electrical permittivity. Energy contributions from the cross-terms mainly consists of seven interactions terms, namely bond–bond, angle–angle, bond–angle, end-bond–torsion, middle-bond–torsion, angle–torsion, and angle–angle–torsion interactions. Take *E_bond-bond_* and *E_angle-torsion_* as an example. *E_bond-bond_* considers the interaction between two bonds with one common atom, and *E_angle-torsion_* considers the interaction between an angle and a torsion. In this paper, we used the particle–particle particle–mesh (PPPM) method to compute long-range Coulomb interactions [33] and set the cut-off distance for both the van der Waals (vdW) and Coulomb interactions at 10 Å [34].

### 2.2. Model Preparation

**Figure 1:**
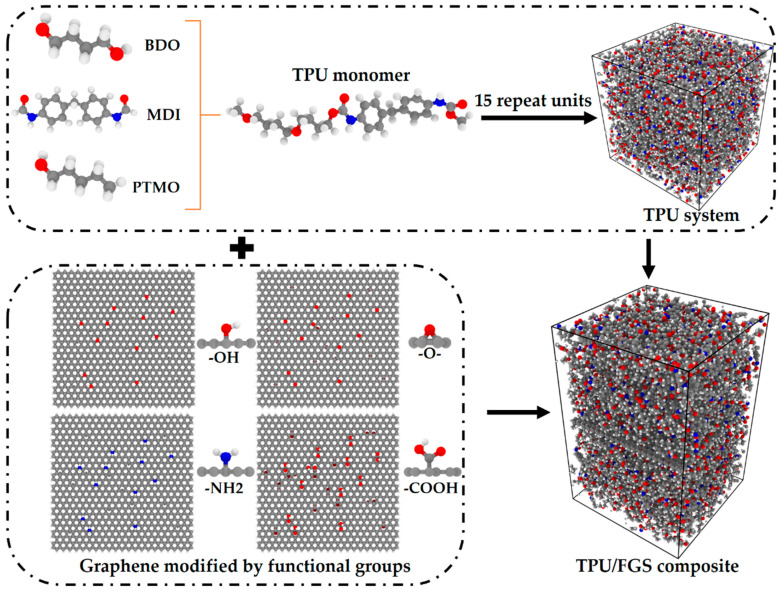
Schematic diagram of TPU/FGS composite models; gray, red, blue, and white balls represent carbon, oxygen, nitrogen, and hydrogen atoms, respectively.

The structure of TPU is considered as a combination of hard segments formed by the reaction of an isocyanate with a chain extender and soft segments formed by polyester or polyether macrodiols. Thus, we selected 4,4′-diphenylmethane diisocyanate (MDI) with butanediol (BDO) as the hard segment of the TPU repeat unit and poly(tetramethylene) oxide (PTMO) as the soft segment, as shown in Figure 1.

To investigate the effect of functional groups on the interfacial properties of the functionalized graphene/TPU nanocomposite, we prepared four kinds of functionalized graphene sheets for the interfacial graphene/polymer models, as shown in Figure 1: hydroxyl (–OH), epoxy (–O-), amine (–NH_2_), and carboxyl (–COOH). Additionally, to study the content of the functional groups on the interfacial properties, each type of functional group was randomly end-grafted to the surface of the graphene sheet at different content values of 2.5%, 5.0%, 7.5%, and 10.0%. The content of functional groups modified on the surface of graphene is defined as the ratio of the number of functional groups to the number of carbon atoms on the surface of graphene.

The neat TPU model was first prepared, and the modified graphene sheet was added subsequently by the Amorphous Cell module in Materials Studio software from Accelrys Software Inc (Dassault Systèmes BIOVIA, San Diego, CA, USA). Considering the effect of the dimension of the simulation system on the accuracy and computational efficiency [14,35,36], we selected 15 TPU chains with 10 repeated units to assemble a TPU matrix, which was followed by the embedding of graphene between the upper and lower TPU matrices with identical conformation, as shown in Figure 1.

### 2.3. Simulation Details

To obtain an equilibrium interfacial model, we applied a conjugate gradient algorithm to minimize the FGS/TPU sandwich system. The model was then put into an NPT ensemble (constant number of atoms, pressure, and temperature) at 500 K and 1 atm for 1000 ps. We chose 0.5 fs as the timestep. Afterward, the system was cooled to 300 K by 50 K decrements for 1000 ps with a 0.5 fs timestep. Finally, the system was relaxed under an NVT ensemble at 300 K for 1000 ps to achieve a zero initial stress state, and sampling data were collected in the last 500 ps. The periodic boundary conditions (PBCs) were introduced in the *x* and *y* directions, and the fixed boundary was set in the *z* direction by placing flat walls on both sides of the simulation in the system. It was worth noting that we set the direction of the non-periodic boundary to the *z* direction due to the limitation of the PPPM method under non-periodic boundary conditions in the LAMMPS. Graphene was treated as a rigid body due to its stiffness and Young’s modulus higher than that of TPU, while the functional groups remained flexible.

## 3. Results and Discussion

### 3.1. Interfacial Energy between TPU and the FGS

**Figure 2:**
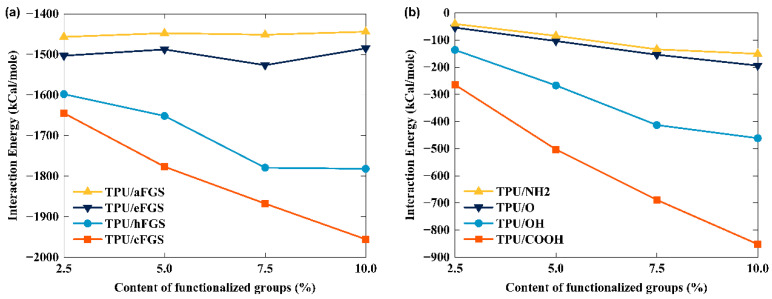
The interaction energy of TPU and (**a**) FGS (**b**) with different functional groups.

To investigate the effect of the types and content of functional groups on the interfacial shear properties between TPU and the FGS, the interfacial strength of the FGS/TPU system should be considered first, because it dominates the interfacial shear properties to some extent. Hence, we calculated the interaction energy of TPU modified with different functional groups (i.e., –OH, –O–, –NH_2_, and –COOH) at a modification density of the graphene surface from 2.5% to 10%, as shown in Figure 2a. The detailed calculation data are provided in Table S1 (Column 1) in the Supplementary Materials. The interaction energy between graphene or the FGS and the polymer matrix was calculated as the difference between the total potential energy of the system and the sum of their potential energy (i.e., the filler phase and the matrix phase), which can be expressed as the following equation:

(17) $$∆E=E_{total}-\left(E_{graphene\;or\;FGS}+E_{polymer}\right),$$

where *E_total_* represents the total potential energy of the polymer/nanocomposite system and *E_graphene or FGS_* and *E_polymer_* represent the potential energy of graphene or the FGS and the polymer matrix, respectively. To easily distinguish the samples, we named different functional-group-modified graphene samples as “nFGS”, where “n” refers to the first letter of the group name. In other words, hFGS denotes the FGS modified with the hydroxyl group (–OH), eFGS denotes the FGS modified with the epoxy group (–O–), aFGS denotes the FGS modified with the amine group (–NH_2_), and cFGS denotes the FGS modified with the carboxyl group (–COOH).

In Figure 2a, regardless of the content of the functional groups, the order of the interfacial interaction energy of the above-mentioned four types of FGS/TPU systems consistently remained as TPU/cFGS > TPU/hFGS > TPU/eFGS > TPU/aFGS. This result corresponds to the order of the polarity of functional groups reported by Zhang et al. [37], suggesting that graphene modified with higher-polarity functional groups (i.e., –COOH or –OH group) can provide stronger interfacial binding energy that that modified with lower-polarity functional groups (i.e., –NH_2_ or –O– group).

Additionally, as the content of functional groups increased from 2.5% to 10.0%, the interaction energy of both TPU/cFGS and TPU/hFGS systems showed a significant increase, which was 11.50% and 18.89%, respectively. In contrast, the variation in the interfacial energy of TPU/eFGS (−1.2%) and TPU/aFGS (−0.87%) could be neglected. It is worth noting that in the process of calculating the interaction energy of TPU and the graphene sheet modified with the latter two functional groups, there were negligible changes in the interaction energy, which failed to show the actual contribution of functional groups to the interfacial binding energy. For this reason, the interaction energy between TPU and each functional group was calculated and compared, as shown in Figure 2b. As expected, the magnitude of the interaction energy between the TPU matrix and functional groups increased with the increasing content of the functional groups from 2.5% to 10%. The exact value was 41.94% for the TPU–OH group model, 38.52% for the TPU–O group model, the 36.72% for TPU–NH_2_ group model, and 45.02% for the TPU–COOH group model. This result comes from the increased number of atoms of functional groups, leading to the increased possibility of interaction between the functional groups and the TPU matrix, which preliminarily indicates that increasing the number of functional groups modified on the graphene surface can play a critical role in the increase in the interaction energy of the TPU/FGS system. In addition, since the order of the interaction energy between the TPU matrix and the functional groups is consistent with that between the TPU matrix and the FGS at a certain content, we further suppose the interaction energy between the two components may be affected by both the polarity and the size of the functional groups. Interestingly, we found that the order of the interaction energy of the TPU/NH_2_ group was smaller than that of the TPU/O group, even though the polarity and size of the two groups were opposite. This phenomenon may be attributed to the LJ potential parameter and charge distribution of the PCFF forcefield. The detailed nonbonded interaction parameter can be found in Table S2.

Nevertheless, we noticed that the slope of the curve corresponding to the variation of the interaction energy decreased with the increase in the content of functional groups. Taking the TPU/OH group as an example, when the OH functional group content modified in graphene gradually increased from 2.5% to 10%, the slope of the curve was 0.957347, 0.546758, and 0.117414, respectively. As a result, the interfacial interaction of the OH group with the TPU matrix tended to be the saturation state with increasing content. A similar consequence for the other three types of samples can be drawn by comparing the variation of slopes tabulated in Table S1. Additionally, by comparing the variation in the interaction energy between TPU and the surface of graphene, as tabulated in Table S1 (Column 3), the interaction energy shows a declining trend for all four different types of samples with increasing content, where the sample with the –COOH group presented a significant decrease among them. This phenomenon could be attributed to the inhibition of TPU matrix adsorption on the graphene surface, due to the excluded volume effect between the functional groups on the graphene surface and the TPU matrix via nonbonded interactions, which depends on the content and size of functional groups [38,39].

**Figure 3:**
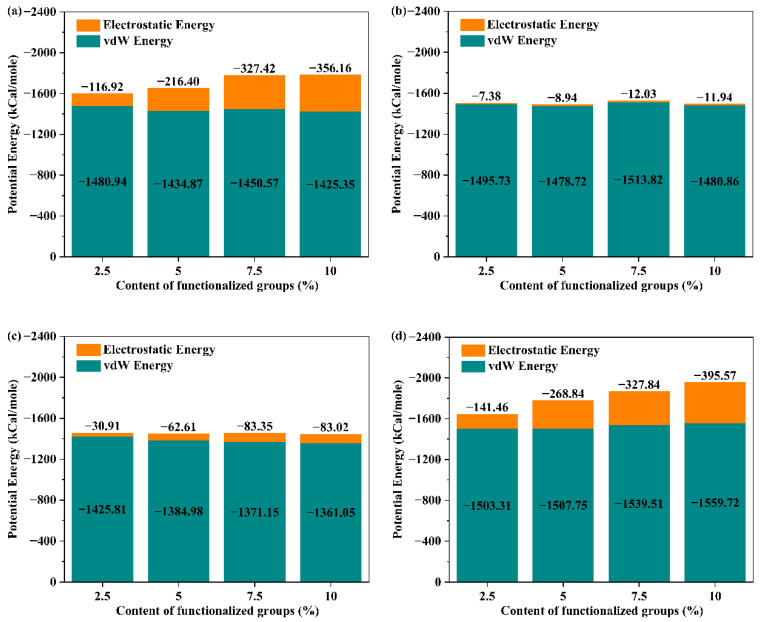
The contribution of electrostatic energy and vdW energy to the interfacial interaction energy of the (**a**) TPU/hFGS system, (**b**) TPU/eFGS system, (**c**) TPU/aFGS system, and (**d**) TPU/cFGS system.

As described in Equation (3), the nonbonded interaction is divided into the sum of the vdW interaction and Coulomb interaction. Hence, in this paper, the amount of interaction energy was obtained by numerically combining the vdW energy and electrostatic energy between the FGS and the TPU matrix. Figure 3 depicts the distribution of vdW energy and electrostatic energy between the FGS and TPU. As shown in Figure 3, the electrostatic energy and its percentage contribution increased as the content of the functional group increased compared to the parts of the vdW energy. Such result is consistent with the trend in Figure 2a, which indicates that the electrostatic interactions between the TPU matrix and functional groups play a key role in affecting the interaction energy of the TPU/FGS system. The formation of hydrogen bonds (H-bonds) between TPU and functional groups also proves this. It can also be observed that the electrostatic energy between TPU and higher-polarity functional groups was much higher than that between TPU and lower-polarity functional groups, which was mainly attributed to the polarity contributing to the formation of hydrogen bonds, thus promoting the interfacial affinity between the FGS and TPU. Unexpectedly, the electrostatic energy of the TPU/hFGS system was close to that of the TPU/cFGS system at the interface. We speculated that the phenomenon may arise from the possibility of both branching groups on the –COOH group to form hydrogen bonds with TPU, leading to a lack of contact sites, which inhibits the forming of H-bonds. To prove the above hypotheses, the effect of the type and content of functional groups on the forming of H-bonds is vital to understand the underlying mechanism.

### 3.2. Hydrogen Bonding

**Figure 4:**
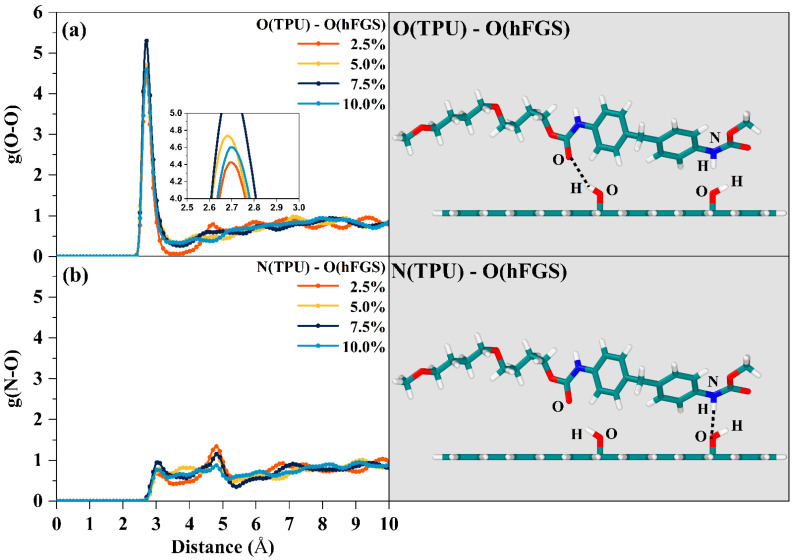
RDF plot of (**a**) O atom of the –C=O group in TPU and O atom of the –OH group in hFGS and (**b**) N atom of the –NH group in TPU and O atom of the –OH group in hFGS.

Although the previous literature reported that the interfacial binding strength between the FGS and TPU can be affected by the polarity of functional groups via H-bond interactions, few studies have reported the explicit relationship of the H-bond donor–acceptor between the TPU matrix and the FGS [40]. In addition, we also noticed that although some reports mention that increasing the functional group content can increase the interfacial strength, the underlying potential mechanism and the effect of the extent of the content on the interfacial strength have not been revealed [41,42]. Thus, to qualitatively investigate the effect of the type and content of functional groups on the forming of H-bonds between the FGS and TPU, as well as clarifying the H-bonds between TPU and the FGS, the radial distribution function (RDF) and the number and number density of H-bonds were introduced. Here, the formula of calculating the RDF is:

(18) $$g\left(r\right)=\frac{1}{\rho 4\pi r^{2}\delta r}\frac{\sum_{t=1}^{T}\sum_{j=1}^{N}∆N\left(r\overset{∆}{\to }r+dr\right)}{N\times T}$$

where *ρ* is the system density (quantity density), *N* is the total number of atoms, *T* is the computation time (steps), and *r* is the radius away from the reference atom. In the process of calculating the RDF, we considered the oxygen atom in the urethane –C=O group as the hydrogen atom acceptor and the nitrogen atom in the urethane NH group as the hydrogen atom donor, which is based on previous experimental studies on the mechanism of hydrogen bond formation between TPUs [43,44,45,46]. Figure 4 and Figures S1–S3 show the radial distribution function of the hydrogen atom donor and acceptor that may form H-bonds between TPU and the FGS modified with –OH, –O–, –NH_2_, and –COOH groups, respectively. To obtain a visual observation of the forming of hydrogen bonds between the TPU matrix and the FGS, we counted the number of H-bonds between TPU and the FGS based on the criterion of hydrogen bonding described in the previous literature that recorded a donor–acceptor distance of less than 3.5 Å and a H acceptor–donor angle of >150° [47,48]. The first peak of the curves in Figure 4 is considered the average distance between the selected two polar atoms in the equilibrium structure, and the height of the curves is believed to be the degree of the aggregation of the two atoms.

**Figure 5:**
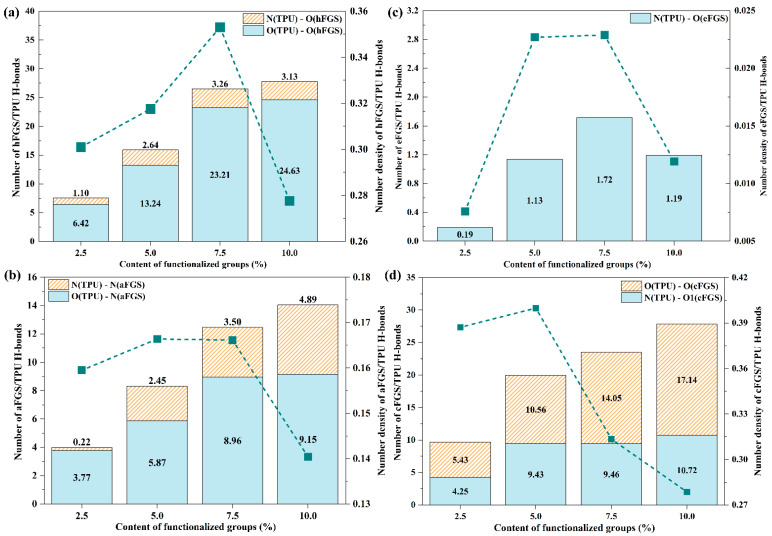
Number of H-bonds in (**a**) hFGS/TPU, (**b**) eFGS/TPU, (**c**) aFGS/TPU, and (**d**) cFGS/TPU systems. Green dotted lines represent the number density of H-bonds.

As shown in Figure 4, the first peak in the RDF of O_hFGS_/O_TPU_ and N_TPU_/O_hFGS_ of TPU/hFGS nanocomposites appeared at 2.61 Å and 3.1 Å, respectively. The peak within 3.5 Å in the RDF is considered the existence of covalent bonds or hydrogen bonds between two atoms, while the peak beyond 3.5 Å is considered the existence of vdW interaction and Coulomb force. It is believed that both O_hFGS_/O_TPU_ and N_TPU_/O_hFGS_ can form H-bonds. In addition, compared to the RDF of O_hFGS_/O_TPU_, a sharper peak at the initial position was observed in the RDF of N_TPU_/O_hFGS_. The above-mentioned result indicates that the average distance between O_hFGS_ and O_TPU_, which may refer to the length of the H-bond, is smaller than that between N_TPU_ and O_hFGS_ and suggests that the possibility of forming H-bonds between O_hFGS_ of the –OH group on the surface of graphene and O_TPU_ of the –C=O group on the TPU chains is higher than that between O_hFGS_ of the –OH group on the surface of graphene and N_TPU_ of the –NH group on the TPU chains. This result is also reflected in the difference in the number of two types of hydrogen bonds, as shown in Figure 5a. As shown in Figure 5, the number of hydrogen bonds formed between O_hFGS_ and O_TPU_ was nearly seven times greater than the number between N_TPU_ and O_hFGS_, which indicates that the first type of hydrogen bond plays a major role in the binding energy.

For the TPU/eFGS model (Figure S1), the first peak of RDF appeared at 3.1 Å, which is within the threshold distance of H-bonds, indicating the existence of H-bonds between the TPU matrix and the –O– group. Nevertheless, the first peak value of N_eFGS_/O_TPU_ in the TPU/eFGS model was about 0.6833, 0.7988, 0.97856, and 0.7832, suggesting that the possibility of forming H-bonds between the –O– group and TPU reaches the maximum with the content of functional groups at 7.5%. As shown in Figure 4 and Figure S1, both the heights of the first peak of the TPU/hFGS model and the TPU/eFGS model reached the maximum value at a content of 7.5% with an increase in the content of functional groups, which means the forming of the H-bonds achieves a saturated state. By comparing the number of H-bonds formed at the interfaces of all TPU/FGS systems, the number of hydrogen bonds formed in the TPU/eFGS model was much smaller than in the other three systems, which indicates that the hydrogen bonds between graphene modified with the –O– group and the TPU matrix are not easily formed and the hydrogen bond strength is also weaker than in the other three systems.

For the TPU/aFGS model (Figure S2), the first peak of the RDF between O_TPU_ in the –C=O group and N_aFGS_ in the –NH_2_ group appeared at around 3.03, while for N_TPU_ in the –NH group and N_aFGS_ in the NH_2_ group, the peak appeared at around 3.1, indicating H-bonds can be formed both between O_TPU_ and N_aFGS_ and between N_TPU_ and N_aFGS_. The value of the first peak of the RDF of O_TPU_-N_aFGS_ decreased significantly from 10 Å to 3 Å with an increase in the functional group content, followed by a slight decrease from 5 Å to 1 Å. By contrast, the value of the first peak of the RDF of O_TPU_-N_aFGS_ remained at a stable value of about 3 at an increase in the content of functional groups from 5% to 10%. This phenomenon indicates the H-bonds between TPU and the aFGS are mainly formed with N_aFGS_ as the H-bond donor and O_TPU_ as the H-bond acceptor at a content of 2.5%, and the possibility of forming H-bonds between NTPU and N_aFGS_ increases as the content of functional groups increases from 5.0% to 10.0%, which corresponds to the result displayed in Figure 5c. By comparing the position and peak of the first peak, it can be concluded that the strength of H-bonds between O_TPU_ and N_aFGS_ is stronger than that between N_TPU_ and N_aFGS_.

For the TPU/cFGS model (Figure S3), the RDF of O_cFGS_-O_TPU_ and NTPU-O1cFGS displayed a sharper and closer peak than that of N_TPU_-O2_cFGS_. The place of the peak of N_TPU_-O2_cFGS_ appeared at 3.8 A, which exceeds the threshold distance of the H-bond, indicating that NTPU cannot form a H-bond with the O of the –OH group in the cFGS. In other words, there may exist two types of H-bonds, i.e., the first type of H-bond contains the –C=O group on the –COOH group (cFGS) as the H-bond acceptor and the –NH group (TPU) as the H-bond donor, and the second type of H-bond contains the –OH group on the –COOH group (cFGS) as the H-bond donor and the –C=O group (TPU) as the H-bond acceptor. Comparing the place of the first peak, the first peak position of the RDF of O_cFGS_-O_TPU_ was smaller than that of N_TPU_-O1_cFGS_, suggesting that the strength of H-bonds formed by the former group is greater compared to the latter group. As shown in Figure S3, the maximum values of the first peak of the RDF of O_cFGS_-O_TPU_ and N_TPU_-O1_cFGS_, i.e., the highest probability of forming H-bonds, were at 2.5% and 5% of the content of functional groups, respectively. This indicates that the content of functional groups has different effects on the forming of H-bonds between the two different functional groups and the TPU matrix, which may be attributed to the polarity of the functional groups. Furthermore, by comparing the sum of the density of H-bonds of the two types at different contents, we found that the density of H-bonds reaches the maximum at a content of 5%, which indicates that the number of H-bonds formed between the cFGS and TPU reaches saturation when the –COOH group content is 5%. It is worth mentioning that the relationship between the number of H-bonds between the two is consistent with the order of electrostatic energy, a phenomenon that exposes the dominant role of H-bonds in the electrostatic energy. It is also observed that the number of H-bonds formed by the former with the O atom of –OH in TPU as the H-bond donor and the O atom of the –C=O group in TPU as the H-bond acceptor is significantly larger than that of the latter, which may be due to the presence of –C=O inhibiting the formation of this type of H-bonds, which verifies the reasonableness of the above hypothesis. Therefore, from the above results, we can conclude that the novelty here is that although increasing the content of functional groups can provide more hydrogen bonds between the interfaces, it cannot improve the strength of H-bond interactions due to the forming of H-bonds achieving a saturation state.

### 3.3. Pull-Out Simulation

**Figure 6:**
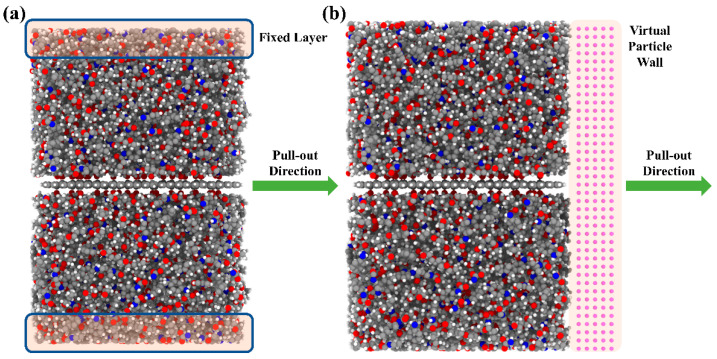
Schematic transverse pull-out simulation model (**a**) without a virtual particle wall and (**b**) with a particle wall.

**Figure 7:**
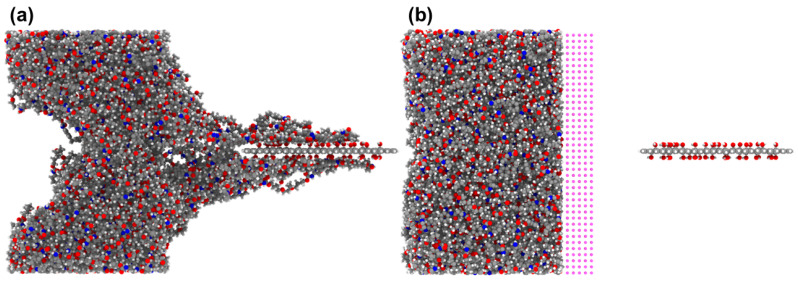
Snapshot of the pull-out displacement of the FGS at 80 Å (**a**) without a virtual particle wall and (**b**) with a particle wall.

**Figure 8:**
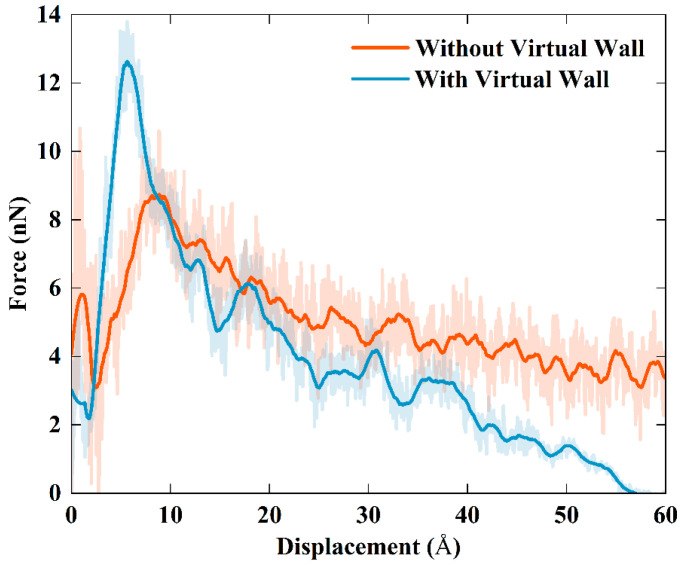
The pull-out force versus displacement with two different simulation schemes (blue line represents the scheme with a virtual wall and red line represents the scheme without a virtual wall).

To investigate the effects of the type and content of functional groups on the interfacial mechanical properties between TPU and the FGS, we performed FGS pull-out simulations in sliding mode. Graphene was pulled out from the TPU interface by applying a constant velocity (0.001 Å/fs). It is worth mentioning that we originally intended to use the method of pulling out graphene from the TPU matrix with the upper and lower ends fixed to characterize the variation in the pull-out force with the type and content of functional groups, as shown in Figure 6a. During the process of pulling out displayed in Figure 7a, it was found that a few TPU molecular chain fragments were dragged out with the graphene pulled away from the TPU matrix by the affinity interaction between the FGS and the TPU matrix. According to the previous literature reported by Jin et al. [49], when graphene containing functional groups is pulled out from the substrate with molecular chains attached to its surface, the original shear surface located at the interface will shift from the interface to the TPU matrix inside, resulting in a decrease in shear force. However, their simulation work did not focus on evaluating the effect of the type and content of functional groups on the interfacial shear properties between TPU and the FGS. The focus of this study lay on the shift from the mechanical interface issues between the filler phase and the matrix phase to the mechanical properties of the matrix itself. Thus, inspired by previous work, we set a virtual wall consisting of the particles along the pull-out direction in the simulation to ensure the position of the relative sliding between the FGS and TPU located at the interface by preventing TPU chains close to the surface from detaching from the FGS, as shown in Figure 6b and Figure 7b. To evaluate the validity of this design, we compared the shear pull-out curves in two different modes, as shown in Figure 8. As shown in Figure 8, the peak of the curve with a virtual wall was larger than that of the curve without a virtual wall, which indicates that the force required to pull the FGS out of the TPU matrix is higher than the force to be overcome when the molecular chains inside the matrix are separated, implying the interfacial shear properties between the TPU matrix and the FGS can be effectively characterized by this simulation approach to some extent.

**Figure 9:**
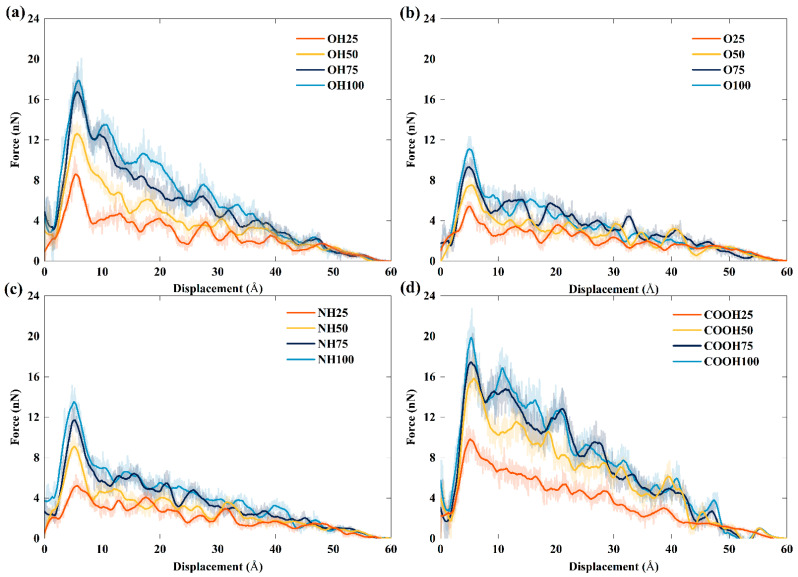
The pull-out force versus displacement of the (**a**) TPU/hFGS model, (**b**) TPU/eFGS model, (**c**) TPU/aFGS model, and (**d**) TPU/cFGS model.

Figure 9 shows the pull-out force versus displacement curve for different types and content of functional groups in sliding mode. The entire pull-out process can be divided into two stages: First, the pull-out force increases sharply to the peak at the initial stage, and then there is a drop in the force in the final stage. Like in the previous findings in the case of the pull-out force [50,51], the increase in the pull-out force is mainly attributed to the elastic deformation of the TPU matrix near the interface and the formation of a new surface, which are caused by the nonbonded interactions between the FGS and TPU during the separation of graphene. When the pull-out force reaches the peak, relative sliding starts to occur at the interface, indicating the peak curve corresponds to the interfacial maximum pull-out force. The phenomenon is similar to the static-friction-to-dynamic-friction transition in stick-slip friction [52], which was also reported by Xia et al. [53]. To quantitatively describe the effect of the type and content of functional groups on the interfacial properties of the FGS/TPU nanocomposite, we considered the interfacial maximum pull-out force as the indicator for comparing the reinforcing role of the discussed functional groups.

**Figure 10:**
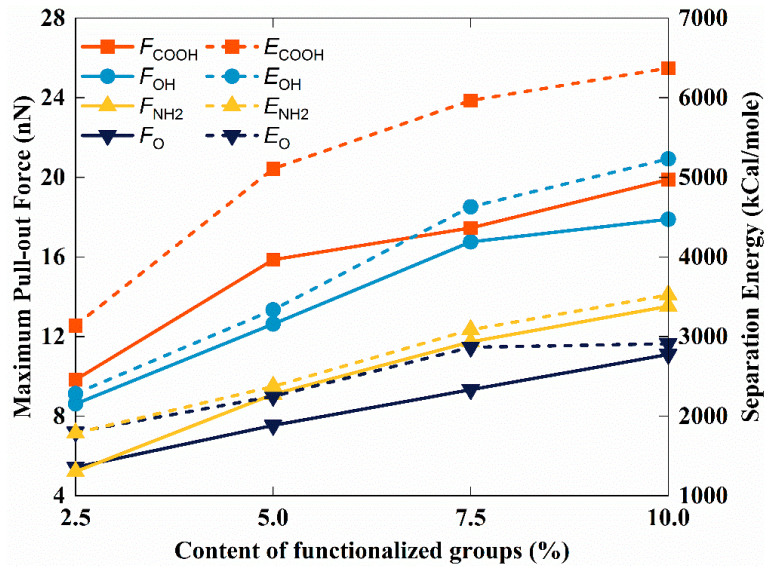
Variation in the maximum pull-out force and separation energy for different types and content of functional groups.

Figure 10 illustrates the effect of the type and content of different functional groups on the maximum pull-out force and separation energy. As shown in Figure 10, the order of the magnitude of the maximum pull-our force was consistent with the order of polarity of the functional groups (–COOH > –OH > –NH_2_ > –O). This phenomenon revealed that the electrostatic interactions between the TPU matrix and the FGS dominate the traction separation behavior of the FGS compared to the mechanical interlocking effect caused by the size of functional groups [54,55], which is one of the novelty points of this work. In addition, we observed that the difference between TPU/hFGS and TPU/cFGS models in the value of the maximum pull-out force is similar to that in the electrostatic energy, as well as that in the number of H-bonds between the TPU matrix and the FGS (i.e., hFGS and cFGS). Therefore, the maximum pull-out force has a positive correlation with the electrostatic interaction and the H-bond interaction, which will be studied in future work. As the content of functional groups increased up to 10.0%, we calculated the increments in the energy required for graphene modified with different types to pull away from the TPU matrix were 61.69% for the TPU/cFGS model, 129.08% for the TPU/hFGS model, 97.02% for the TPU/aFGS model, and 102.99% for the TPU/eFGS model. This result shows that interfacial shear properties can be enhanced significantly by modifying the –COOH group or –OH group on the surface of graphene, where the FGS modified with the –COOH group shows better performance.

## 4. Conclusions

In this work, the effects of the type and content of functional groups modified on the surface of graphene on the interfacial shear properties of functionalized graphene sheet/TPU nanocomposites were investigated by calculating the interaction energy between TPU and the FGS with different types and contents of functional groups and performing pull-out simulations in sliding mode. The RDF, the number of H-bonds, and the number density of H-bonds were calculated to reveal the relationship between the interfacial binding strength and the H-bond interaction between the TPU matrix and the FGS modified by different types and contents of functional groups. In addition, we selected to set a virtual particle wall in front of the TPU matrix instead of fixing the upper and lower ends of the TPU matrix to capture the traction-separation behavior at the interface between the TPU matrix and the FGS during the pull-out process. Based on the MD results, the following conclusions are drawn:

1. The interfacial shear resistance of TPU/hFGS and TPU/cFGS models is higher than that of TPU/aFGS and TPU/eFGS models, which is due to the stronger electrostatic interaction and H-bond interactions between TPU and functional groups with higher polarity, such as the –COOH or the –OH group. In addition, during the process of pulling out, the electrostatic interaction and H-bond interactions dominate the traction-separation behavior of the FGS compared to the mechanical interlocking effect caused by the size of functional groups.
2. According to the RDF analysis, the TPU matrix has a larger possibility to form H-bonds with the –OH, –NH2, and –COOH groups than with the –O– group. Except for the inability to form hydrogen bonds between the O atoms of the –NH group of the TPU matrix and the –OH group of the cFGS due to the limitation of contact sites between the TPU matrix and the –COOH group, all the former three functional groups mentioned can form two types of H-bonds with the TPU matrix through nonbonded interactions between their polar atoms. This also leads to the reduced probability of forming H-bonds between the TPU matrix and the cFGS, which is reflected in the electrostatic energy, and the total amount of H-bonds between TPU and the cFGS are slightly higher than those between TPU and the hFGS, while the number of H-bonds formed between the –OH group of the cFGS and the TPU matrix is lower than that between the –OH group of the hFGS and the TPU matrix.
3. With the increase in the functional group content, the hydrogen bond formation between TPU and the FGS tends to a saturation state.

Although our work has provided some understanding of and insights into the interfacial mechanical properties between the TPU matrix and the FGS modified with different types and contents of functional groups from a microscopic perspective, in the actual preparation process of functionalized graphene, both the surface and the side of graphene can be modified by more than two types of functional groups, which will be discussed in our future work. In addition, due to the limitation of the microscopic simulation system, there is still a discrepancy between the accuracy of the simulation results we obtained and the actual; therefore, a multi-scale research platform will also be built for the study of the interface properties in future work.

## Data Availability

Not applicable.

## Supplementary Materials

The following supporting information can be downloaded at: https://www.mdpi.com/article/10.3390/polym14225032/s1, Table S1: The interaction energy of TPU with FGS and its each part; Table S2: Nonbond Interaction Parameters; Figure S1: RDF plot of N atom of –NH group in TPU and O atom of –O- group in eFGS; Figure S2: RDF plot of (a) O atom of C=O group in TPU and N atom of NH2 group in aFGS (b) N atom of –NH group in TPU and N atom of NH2 group in aFGS; Figure S3: RDF plot of (a) O atom of C=O group in TPU and O atom of OH group in cFGS (b) N atom of –NH group in TPU and O atom of C=O group in cFGS (c) N atom of –NH group in TPU and O atom of OH group in cFGS.
