# Supplementary material for: Molecular Dynamics Simulation of the Interfacial Shear Properties between Thermoplastic Polyurethane and Functionalized Graphene Sheet
Source: Polymers (Basel). 2022 Nov 20;14(22):5032. doi: 10.3390/polym14225032 (PMC9694644; doi:10.3390/polym14225032)
Supplement: Supplementary file 1 [file polymers-14-05032-s001.zip › polymers-2037674-supplementary.pdf]

Supplementary Materials

Table S1: The interaction energy of TPU with FGS and its each part

Model	ΔE_{tot} (kCal/mole)	ΔE_{fg} (kCal/mole)	ΔE_{pg} (kCal/mole)*
OH25	-1597.8091	-136.3177	-1461.4914
OH50	-1651.3662	-266.8211	-1384.5451
OH75	-1779.0537	-412.7077	-1366.3461
OH100	-1781.5958	-461.1655	-1320.4303
O25	-1503.1035	-53.8817	-1449.2218
O50	-1487.6606	-103.4461	-1384.2145
O75	-1526.8296	-153.7790	-1373.0506
O100	-1484.8994	-193.7762	-1291.1233
NH25	-1456.6676	-40.4411	-1416.2265
NH50	-1447.4916	-84.3402	-1363.1514
NH75	-1451.2858	-133.9499	-1317.3360
NH100	-1443.9901	-150.5562	-1293.4340
COOH25	-1644.7305	-264.4425	-1380.2880
COOH50	-1776.6160	-502.8351	-1273.7809
COOH75	-1867.3655	-688.6883	-1178.6772
COOH100	-1955.3862	-851.8146	-1103.5716

* ΔE_{tot} denotes the total interaction energy, ΔE_{fg} denotes the interaction energy between TPU and functional groups, ΔE_{pg} denotes the interaction energy between TPU and pure graphene

Table S2: Nonbond Interaction Parameters

i	σ_i (Å)	$\mathcal{E}_i$ $(kcal/mol)$
C2	4.010	0.054
Hc	2.995	0.020
O_2	3.420	0.240
C_2	3.810	0.120
N_2	3.600	0.106
Cp	4.010	0.064
Hn2	1.650	0.013
O_1	3.300	0.013
Oc	3.535	0.240
C3	4.010	0.054
Ho2	1.110	0.013
C_1	3.810	0.120
Ho	1.098	0.013
Hn	1.098	0.013
Nb	4.070	0.065
O3e	3.535	0.240
Oh	3.535	0.240

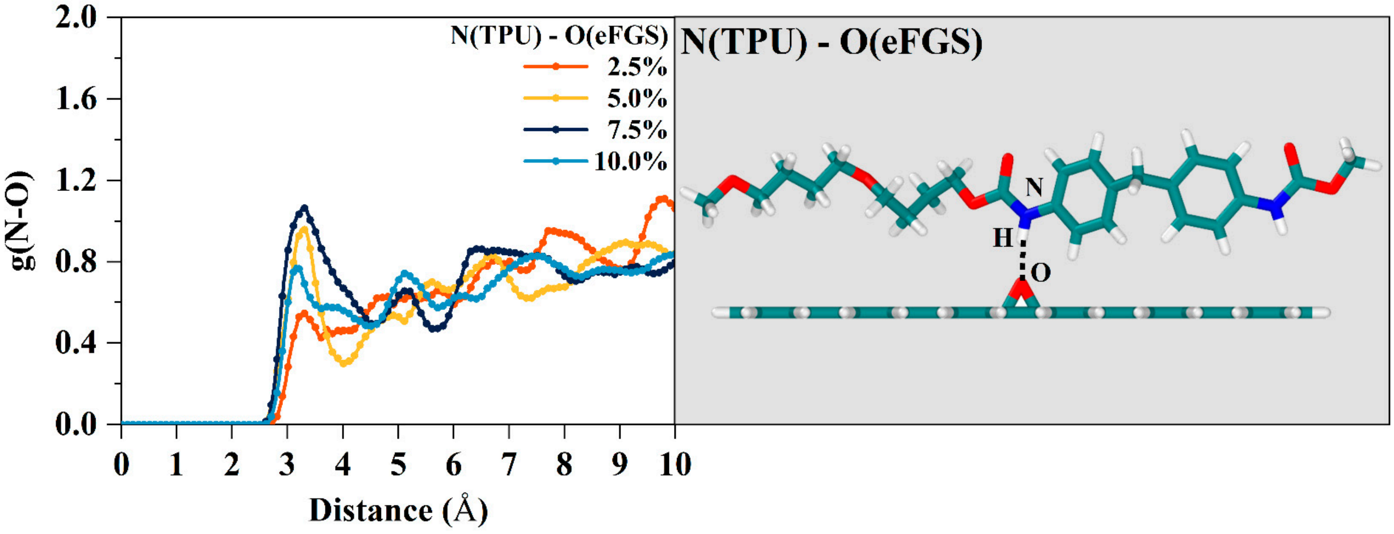

Figure S1: RDF plot of N atom of –NH group in TPU and O atom of –O- group in eFGS

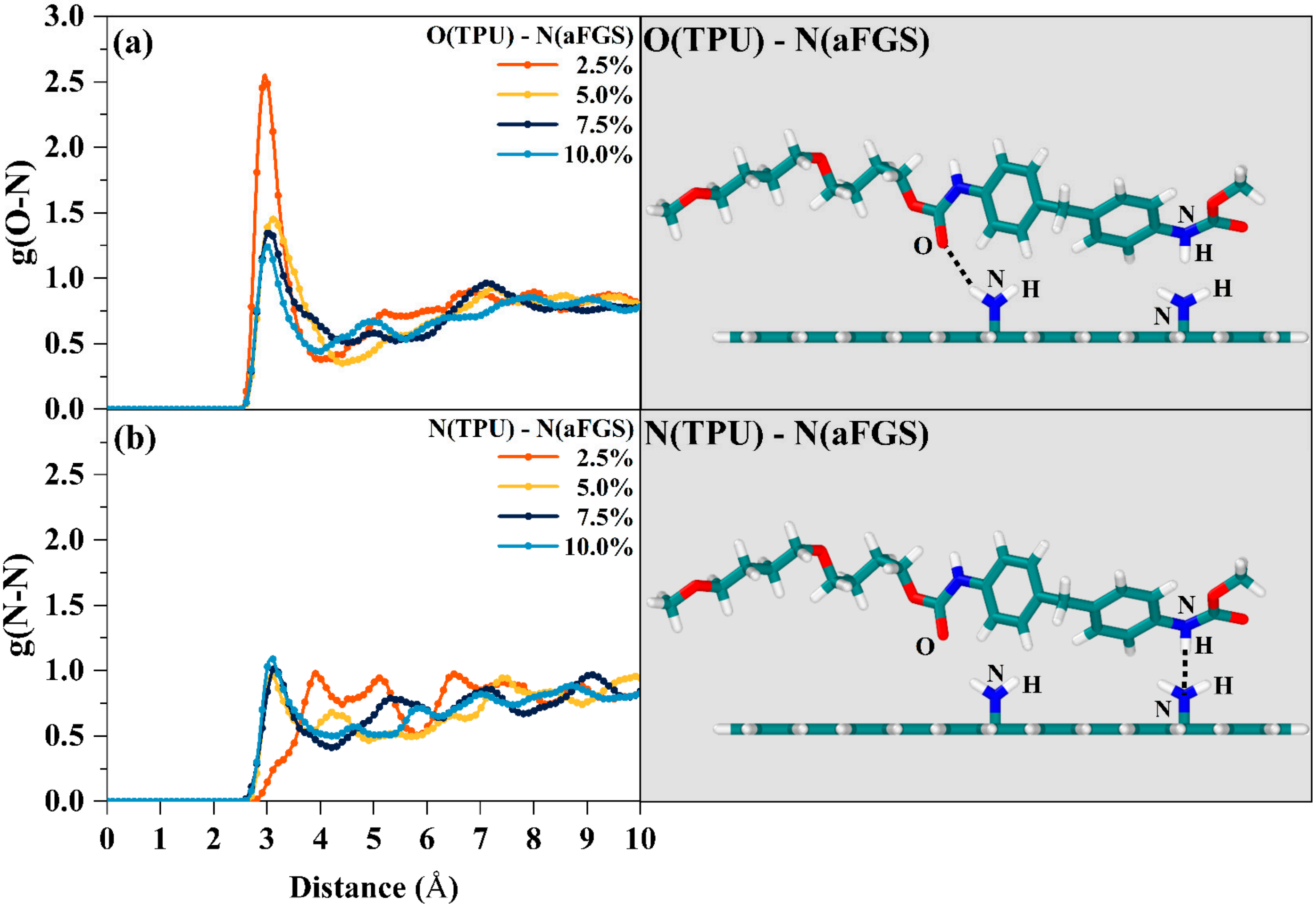

Figure S2: RDF plot of **(a)** O atom of C=O group in TPU and N atom of NH2 group in aFGS **(b)** N atom of –NH group in TPU and N atom of NH2 group in aFGS

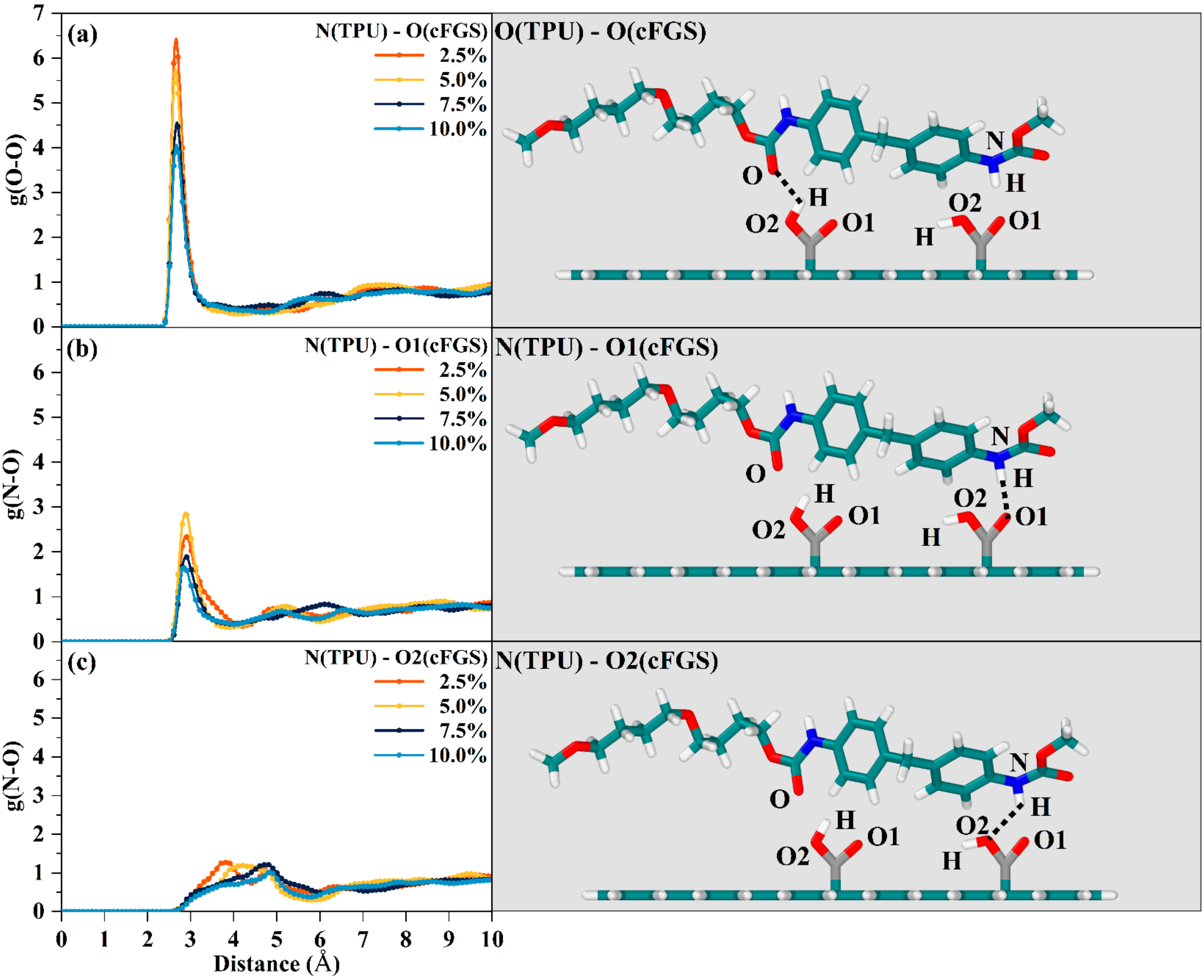

Figure S3: RDF plot of **(a)** O atom of C=O group in TPU and O atom of OH group in cFGS **(b)** N atom of –NH group in TPU and O atom of C=O group in cFGS **(c)** N atom of –NH group in TPU and O atom of OH group in cFGS
